# Machine Learning Descriptors for Data‐Driven Catalysis Study

**DOI:** 10.1002/advs.202301020

**Published:** 2023-05-16

**Authors:** Li‐Hui Mou, TianTian Han, Pieter E. S. Smith, Edward Sharman, Jun Jiang

**Affiliations:** ^1^ Hefei National Research Center for Physical Sciences at the Microscale School of Chemistry and Materials Science University of Science and Technology of China Hefei Anhui 230026 China; ^2^ Hefei JiShu Quantum Technology Co. Ltd. Hefei 230026 China; ^3^ YDS Pharmatech ETEC 1220 Washington Ave. Albany NY 12203 USA; ^4^ Department of Neurology University of California Irvine CA 92697 USA

**Keywords:** catalytic descriptors, heterogeneous catalysis, high‐throughput experiments, machine learning, theoretical simulations

## Abstract

Traditional trial‐and‐error experiments and theoretical simulations have difficulty optimizing catalytic processes and developing new, better‐performing catalysts. Machine learning (ML) provides a promising approach for accelerating catalysis research due to its powerful learning and predictive abilities. The selection of appropriate input features (descriptors) plays a decisive role in improving the predictive accuracy of ML models and uncovering the key factors that influence catalytic activity and selectivity. This review introduces tactics for the utilization and extraction of catalytic descriptors in ML‐assisted experimental and theoretical research. In addition to the effectiveness and advantages of various descriptors, their limitations are also discussed. Highlighted are both 1) newly developed spectral descriptors for catalytic performance prediction and 2) a novel research paradigm combining computational and experimental ML models through suitable intermediate descriptors. Current challenges and future perspectives on the application of descriptors and ML techniques to catalysis are also presented.

## Graphical abstract

This review briefly summarizes the tactics for the utilization and extraction of descriptors in machine Learning (ML)‐assisted experimental and theoretical catalysis research. The effectiveness and advantages as well as limitations of various descriptors are discussed. The newly developed spectral descriptors for catalytic performance prediction and a novel research paradigm combining computational and experimental ML models through suitable intermediate descriptors are highlighted.

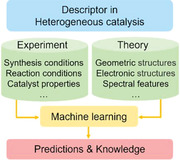

## 1 Introduction

Catalysis plays an important role in modern chemical industry, with its many chemical processes—such as energy conversion and pollutant removal—that need catalysts to greatly reduce input costs and increase product yields.^[^ ^1^ ^]^ Identifying optimal reaction conditions, designing efficient catalysts, and revealing catalytic mechanisms are important research areas in the field of catalysis. Experimental trial‐and‐error is the classical research paradigm, in which one variable is usually evaluated at a time, incurring the disadvantages of long timelines and low efficiency. Moreover, traditional experimental and computational methods rely heavily on prior knowledge and are vulnerable to human cognitive biases. With the development of computational chemistry, theoretical simulations mainly based on density functional theory (DFT) calculations that incorporate simplified model systems can capture the critical aspects of complex realistic systems and thus guide the rational design of experiments.^[^ ^2^ ^]^ However, the dramatic increase in computational cost incurred as the complexity of model structures increases poses a great challenge to this approach.

**Figure 1:**
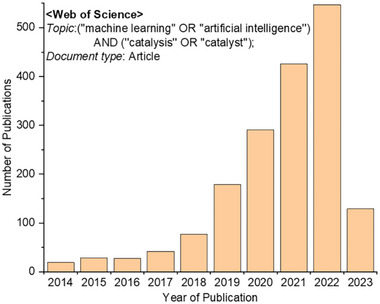
Number of recent publications obtained by searching the topics “machine learning/artificial intelligence” and “catalysis/catalyst” in the Web of Science database.

Machine learning (ML) is a branch of artificial intelligence that is capable of dramatically lowering this computational cost. It can learn from existing data and generate a training model for predicting results outside of the training dataset, thus providing a promising approach for accelerating catalysis research progress.^[^ ^3^ ^]^ Indeed, the recent trend of publications in ML‐based catalysis research obtained from the Web of Science database (**Figure** **1**) indicates that ML is being utilized increasingly in this field. Some excellent reviews have summarized relevant works in catalyst design and discovery and also have discussed the importance of the choice of suitable descriptors (also called features).^[^ ^4^ ^]^ Catalytic descriptors are representations of reaction conditions, catalysts and reactants, that are extracted from original data to describe target properties (e.g., yield, selectivity, adsorption energy, energy barriers, etc.) in a machine‐recognizable form.^[^ ^5^ ^]^ Although the choice of ML algorithm is very important, descriptor definitions play a decisive role in the prediction accuracy of ML models, while algorithm optimization can only bring the models as close to the upper limit of accuracy as otherwise possible. Moreover, a key to the rational design of high‐performance catalysts is understanding quantitative structure–activity relationships, which correlate catalytic descriptors with desired catalytic behavior. Therefore, mining effective descriptors from the training data is a crucial step in ML‐assisted catalysis research. Most previous reviews focused primarily on theoretical catalysis and related descriptors.^[^ ^6^ ^]^ However, this review will provide a comprehensive overview of the utilization and extraction of descriptors derived from both experimental data and theoretical calculations. One of its aims is to stimulate thinking about how to use intermediate descriptors to combine experimental and computational ML models so as to improve accuracy and efficiency. We start with the introduction of common descriptors used in ML models that can be trained on conventional or high‐throughput experiments or published data. Then, the relatively plentiful descriptors extracted from computational datasets are discussed, including their effectiveness and advantages as well as their limitations. We highlight newly developed spectroscopic descriptors for catalytic performance prediction and a promising research paradigm combining large theoretical and small experimental data sets through suitable intermediate descriptors.

## 2 Experiment‐Based Descriptors

In the 1990s, ML techniques were shown to possess high efficiency and accuracy when applied to experimental catalysis research.^[^ ^7^ ^]^ Kito and coworkers employed an artificial neural network (ANN) to learn experimental data to predict the selectivity of oxidative dehydrogenation of ethylbenzene on promoted and unpromoted SnO_2_ catalysts. They used descriptors corresponding to the nature of catalysts including the presence of unusual valence, the amount and surface area of the catalysts, typical valence, ionic radius, coordination number, electronegativity, partial charges of oxygen ions, and standard heat of formation of oxides. The selectivities of various products such as styrene, benzaldehyde, CO, and CO_2_ were set as target properties.^[^ ^7a^ ^]^ Their prediction results suggested that, compared to a knowledge‐based expert systems approach, ANN requires less effort and labor and gives much better predictions. Since catalytic reactions on heterogeneous surfaces are very complex, catalytic performance is usually determined by a multitude of factors. Therefore, in some cases, additional factors including synthesis variables, operating conditions, and reaction conditions are also used as descriptors in experimental ML models.^[^ ^8^ ^]^ The relative significance of experimental factors for determining catalytic performance can be estimated by descriptor importance analysis, which can help researchers design new experiments more effectively by narrowing down the search space. Taking tree‐based models as an example, importance is determined by reviewing the generated decision trees to determine descriptor prominence and frequency during the decision process.

**Figure 2:**
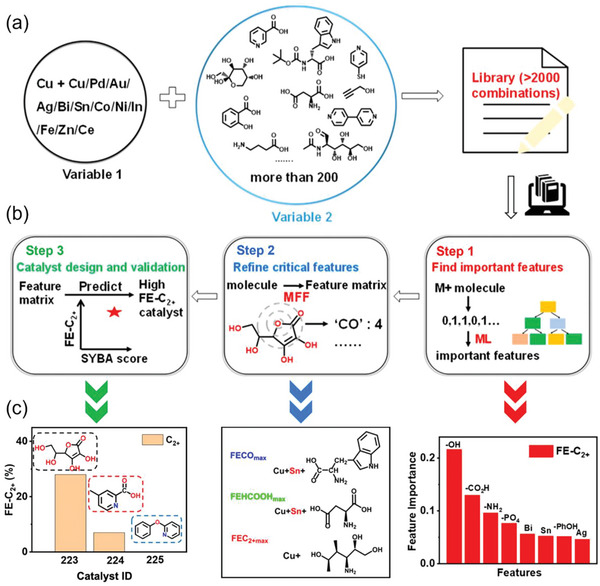
a) An additive library that includes 12 metal salts (Variable 1, M) and more than 200 organic molecules (Variable 2) used to construct a set of CO_2_ reduction electrocatalysts. b) The learning loop consisting of three iterative cycles of “experimental test–ML analysis–prediction and redesign” to accelerate the search for high‐performance catalysts. c) Feature importance of FE‐C_2+_ obtained by gradient boost decision tree regressor analysis in the first round of learning (right), the selected catalysts with maximal FE for different products after the second round of learning (middle), and the experimentally‐measured FE‐C_2+_ values of three selected catalysts (left). FE = Faradaic efficiency; MFF = molecular fragment featurization. Reproduced with permission.^[^ ^11^ ^]^ Copyright 2021, American Chemical Society.

In addition to comprehensively describing a catalytic reaction using different dimensions and types of descriptors, ingenious utilization of these descriptors is very important for guiding the design of experiments. Copper catalysts are widely used in the electrochemical CO_2_ reduction reaction (CO_2_RR) for producing C_1_ species (e.g., CO, HCOOH, CH_4_, and CH_3_OH) and C_2+_ species (e.g., C_2_H_4_, C_2_H_6_, C_2_H_5_OH, and C_3_H_7_OH).^[^ ^9^ ^]^ Subtle changes in morphology and surface structure of such catalysts can be induced by a very wide range of additives during catalyst preparation, leading to significant changes in product selectivity.^[^ ^10^ ^]^ The lack of clear structure–property relationships associated with such a large number of possible additives renders the selection of optimal additive combinations difficult. To solve this problem, Guo et al. developed a strategy incorporating three rounds of learning that combines experimental results and ML,^[^ ^11^ ^]^ and applied it to an additive library of 12 metal salts and 200 organic molecules. Presence of a metal and/or functional organic group was used to define input features of a catalyst recipe for predicting its activity and selectivity. Starting from analysis of a representative fraction of the more than 2000 combination recipes from this library (**Figure** **2**a), their method iteratively determined the effects on catalyst selectivity of a particular feature and of feature combinations, and finally identified catalysts individually selective for generating predominantly CO, HCOOH or C_2+_ products (Figure 2b). In the first round of learning, one‐hot vectors of additives were combined to form descriptors, each showing the presence or absence of a given metal or functional group in a recipe (Figure 2b, right). The faradaic efficiencies (FE) of various products were the outputs. Both qualitative classification algorithms—such as decision tree, random forest, and XGboost^[^ ^12^ ^]^—and quantitative regression algorithms—such as linear regression, the least absolute shrinkage and selection operator,^[^ ^13^ ^]^ and gradient boost decision tree regressor^[^ ^14^ ^]^—were used to predict target properties, so as to identify which features were most critical for selectivity. The results showed Sn to be the most significant and positive metal additive for FE‐CO, and the aliphatic OH group on an organic molecule to be the most important feature for FE‐C_2+_ (Figure 2c, right). To further refine these critical features, in the second round of learning, the local structure of the organic molecule was transformed by molecular fragment featurization (MFF)^[^ ^11^, ^15^ ^]^ into a feature matrix to be used as a descriptor set (Figure 2b, middle). Classification and regression analyses suggested that besides the importance of Sn for FE‐CO, as found in the first round of learning, presence of a nitrogen heteroaromatic ring in an organic molecule favors generating CO while an aliphatic amino group favors generating HCOOH. Aliphatic amine presence was shown to have a positive effect on FE‐C_2+_, as confirmed by the activity of the catalyst prepared with sorbitol as the organic additive (Figure 2c, middle). In the third round of learning, the authors used a “random intersection tree”^[^ ^16^ ^]^ to examine important variable combinations that have a positive or negative synergistic effect on catalytic selectivity. The results showed that combinations of an aliphatic hydroxyl group with aliphatic carboxylic acids, an aliphatic amine or aliphatic ammonium salts tend to enhance FE‐C_2+_, while combinations of aromatic rings, aromatic carboxylic acids and nitrogen‐containing heterocycles tend to reduce FE‐C_2+_. Following this finding, 24 molecules were designed and the corresponding FE‐C_2+_ was assessed by a voting regressor. Scores of synthetic Bayesian accessibility (SYBA)^[^ ^17^ ^]^ were also calculated to predict whether the designed molecules were easy to synthesize (Figure 2b, left). Three commercially available molecules predicted to have high, medium or low FE‐C_2+_ values were experimentally tested, and found to produce FE‐C_2+_ values of 28%, 7% and 0% (Figure 2c, left), respectively, confirming the ML prediction. This study demonstrated the potential of ML to accelerate catalyst design by efficiently extracting information from a limited amount of experimental data.

Currently, the development of ML‐assisted catalysis research is largely limited by the scale and quality of experimental data. High‐throughput experimentation can produce large and consistent datasets—which have less variability than conventionally generated data—and is beneficial to the establishment of ML models.^[^ ^18^ ^]^ Nguyen et al. developed a high‐throughput screening instrument that enables the rapid, large‐scale measurement of changes in product composition produced by widely varying catalytic reaction conditions maintained in a well‐defined, process‐consistent manner.^[^ ^18a^ ^]^ They exploited this instrument to automatically evaluate the performance of 20 catalysts under 216 reaction conditions and obtained a data set comprised of 12 708 data points. Such a catalyst data set covering a parametric space of both catalysts and process conditions proved to be essential for understanding how to optimize catalysts and processes cooperatively to improve the selectivity of target products. In this process, the selected descriptors encompassing the information of both catalyst design and experimental process conditions were vital for the accurate prediction of catalytic performance. In addition to generating large datasets containing tens of thousands of data points, the high‐throughput screening instrument can also be used to provide a bias‐free dataset based on random sampling over a huge material space. The power of bias‐free datasets in finding novel catalysts as well as generating catalyst design guidelines has been demonstrated.^[^ ^19^ ^]^

**Figure 3:**
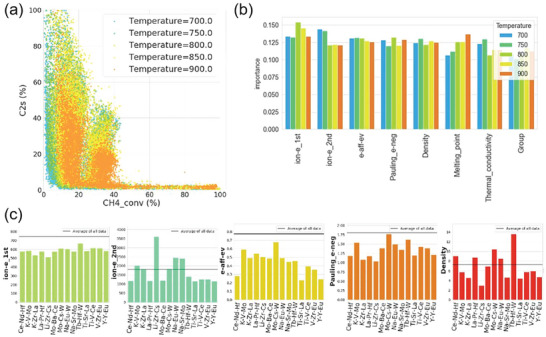
a) C_2_ selectivity versus CH_4_ conversion of the oxidative coupling of methane obtained by high‐throughput experiments. The color represents the temperature. b) Importance analysis by random forest classification. c) Average density, electronegativity, electron affinity, and first and second ionization energies of predicted high‐C_2_‐selectivity catalysts. In each plot, a horizontal line represents the descriptor average over all data for comparison. ion‐e_1st = first ionization energy; ion‐e_2nd = second ionization energy; e‐aff‐ev = electron affinity; Pauling_e‐neg = Pauling electronegativity; Group = periodic table group. Reproduced with permission.^[^ ^18c^ ^]^ Copyright 2022, American Chemical Society.

The underlying factors regulating catalytic performance can also be revealed by employing ML tools to learn from a large high‐throughput experimental dataset. Ishioka et al. explored descriptors for product selectivity in the oxidative‐coupling‐of‐methane (OCM) reaction using ML and physical quantities derived from the periodic table.^[^ ^18c^ ^]^ The large catalyst data set generated from high‐throughput experiments contained 31 334 data points, spanning a variety of process conditions (e.g., temperature and gas flow), and support and catalyst compositions. The relationship between the measured conversion of CH_4_ (CH_4__conv) and selectivity of C_2_H_4_/C_2_H_6_ (C_2_s) as a function of reaction temperature was visualized using a scatter plot (**Figure** **3**a). An unsupervised ML algorithm—hierarchical clustering—first classified data at each temperature into three groups based on dendrogram distance, where C_2_s was used as the objective variable. Three selectivity classification groups (low, medium, and high C_2_s) were then treated as objective variables for supervised classification modeling. Nineteen physical quantities were selected as descriptors for determining the relationship between these quantities and catalyst C_2_s selectivity. Two classifiers—random forest classifier (RFC)^[^ ^13^ ^]^ and support vector classifier (SVC)^[^ ^20^ ^]^—produced accurate predictions with cross‐validation scores of 0.67–0.84. Feature importance analysis by RFC disclosed 5 highly important descriptors for predicting C_2_s: first and second ionization energies, electron affinity, Pauling electronegativity, and density (Figure 3b). In the next step, these 5 most important descriptors and the trained RFC and support vector classifier (SVC) models were used to predict catalysts having high C_2_s from the 62 196 catalysts that were created through permutations of three elements available from the 71 elements. By including support composition identities as additional descriptors, the ML models predicted that 3 previously unreported catalysts (Ti‐V‐Ce‐BaO, Y‐Y‐Eu‐TiO_2_, and La‐Pr‐Hf‐BaO) would have high C_2_s values, as was then validated experimentally. Finally, the authors investigated the physical meaning of the 5 descriptors. For the set of three‐metal catalysts with predicted high‐C_2_‐selectivity, values for each of the 5 important descriptors are shown (Figure 3c); for each descriptor, a line denoting the descriptor average over all three‐metal combinations is shown for comparison. It can be concluded that catalysts predicted to have high‐C_2_s selectivities tend to have low first ionization energies, electron affinities and electronegativities, while their high second ionization energies and densities are comparable to the averages. This study demonstrated the feasibility of using fundamental physical quantities as alternative catalytic descriptors for designing heterogeneous catalysts.

**Figure 4:**
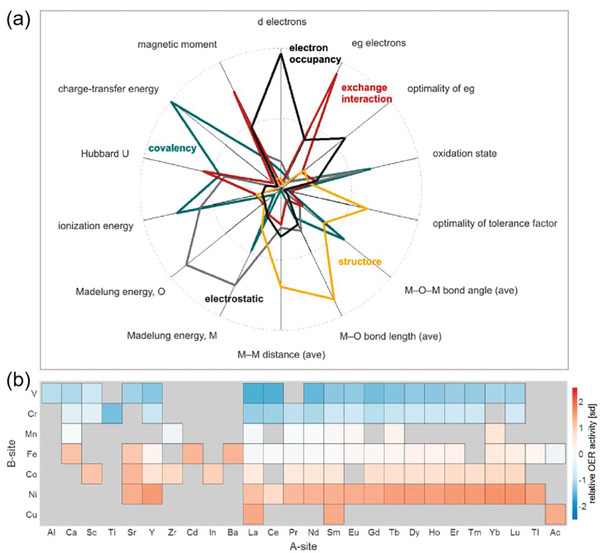
a) Five descriptor families are obtained by factor analysis for 14 descriptors. Larger radial component indicates larger contribution of a descriptor to the factor. The factors can be considered as descriptor families, related to covalency (green), electrostatics (gray), structure (yellow), exchange interaction (red) and electron occupancy (dark gray). b) Heatmap of relative OER activity predictions for ABO_3_ perovskites using the least angle regression model and data mined from the Materials Project database. Warmer colors indicate higher relative OER activity. Reproduced with permission.^[^ ^22^ ^]^ Copyright 2015, American Chemical Society.

To avoid the high cost of high‐throughput experiments, obtaining a large data set from published papers has been an effective approach for mining catalytic descriptors.^[^ ^21^ ^]^ Hong et al. collected 101 oxygen evolution reaction (OER) activities of 51 perovskites (ABO_3_, where A is a rare‐earth or alkaline‐earth metal and B is a transition metal) from previous works and their experimental measurements.^[^ ^22^ ^]^ Fourteen descriptors that may control the surface metal–oxygen bonding were explored in terms of their relationships and predictive power using several statistical approaches including factor analysis^[^ ^23^ ^]^ and linear regression models. Factor analysis demonstrated that these 14 descriptors could be classified into five descriptor families, associated with the oxide's metal–oxygen covalency, electrostatics, structure, exchange interactions and transition‐metal electron occupancy (**Figure** **4**a). Among them, electron occupancy and covalency have the dominant influences on OER activity. However, to obtain the best predictive models, a linear combination of nine descriptors was necessary, demonstrating the importance of considering multiple descriptors in predicting OER activity. Based on the important descriptors so identified, they performed a large‐scale screening of relative OER activities of ABO_3_ perovskites from the Materials Project database.^[^ ^24^ ^]^ Figure 4b shows the heatmap of predictions generated by the best‐performing least angle regression model. It can be seen that higher activities occur for late‐transition metal oxides, and isovalent substitutions of the A‐site cation have a moderate influence on relative OER activity. Günay et al. also carried out a series of ML analyses on published data of important catalytic reactions for the purpose of extracting additional knowledge from them.^[^ ^25^ ^]^ By using catalyst preparation variables, operating variables and reaction conditions as descriptors for the prediction of catalytic performance, the authors determined the effects and relative significance of these features and predicted the outcome of unstudied conditions. These studies provide a promising approach for extracting effective catalytic descriptors that can be used to rapidly screen OER electrocatalysts across a wide chemical space.

To summarize, by applying descriptor‐based ML techniques, great progress has been made in experimental catalysis research. Readily available or economically‐generated parameters are usually used as catalytic descriptors for experimental ML models; these include synthesis conditions, reaction conditions, and physical quantities of catalysts obtained from periodic trend data or materials databases. However, this field is still in its infancy due to the time and cost of obtaining experimental data. Moreover, although information from structural characterization of catalysts and reaction intermediates might be more useful, it is very expensive and sparse, limiting the application of ML and making the exploration of catalytic mechanisms at an atomic level challenging.

## 3 Theory‐Guided Descriptors

Theoretical simulations can provide rich geometric and electronic information for developing catalytic descriptors that are largely beyond the simple observables derived from experiments. For theoretical models of catalysis, the activity of catalysts is usually evaluated by reaction and activation energies on the potential energy surface; catalytic mechanisms can also be unveiled by analyzing the reaction path, charges, free energies, and so on. Moreover, application of Brønsted–Evans–Polanyi (BEP) relations that link activation barriers with reaction enthalpies and scaling relationships between adsorption energies of reaction intermediates have greatly simplified DFT computations.^[^ ^26^ ^]^ These methods enable direct extrapolation of activity trends from adsorption energies of key species, thus avoiding time‐consuming activation barrier calculations and reaction path analyses. Although adsorption energy is an important descriptor of catalytic activity, it is difficult to assess experimentally, making the design of catalysts directly based on it impractical. Descriptors that are simple and easy to compute or measure are being actively explored to correlate adsorption energy and thus catalytic activity. This process can be accelerated by ML models for which adsorption energy is an important prediction target.

### 3.1 Intrinsic Atomic Property Descriptors

**Figure 5:**
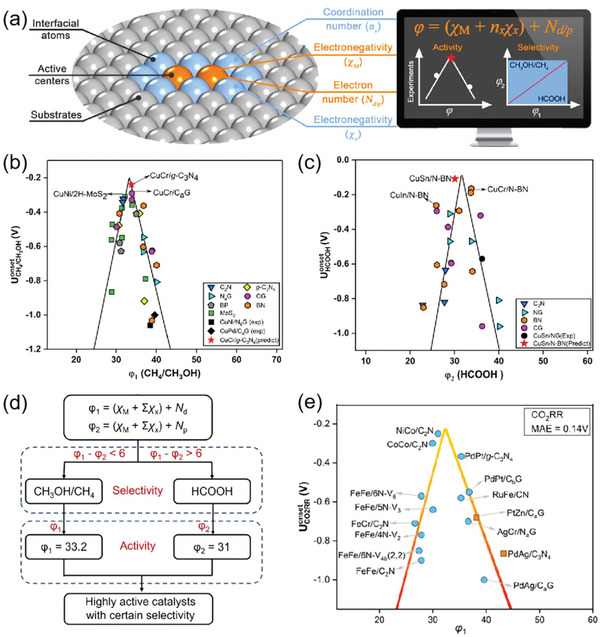
a) Relation between catalyst structure and the proposed descriptor pair *φ*_1_ and *φ*_2_ (for symbol definitions, see text). Volcano plot for b) $U_{\mathrm{CH}4/\mathrm{CH}3\mathrm{OH}}^{\mathrm{onset}}$ versus the descriptor *φ*_1_ and for c) $U_{\mathrm{HCOOH}}^{\mathrm{onset}}$ versus the descriptor *φ*_2_. d) Design/screening procedure for highly active DACs@2D's that possesses high product selectivity for the CO_2_RR using *φ*_1_ and *φ*_2_ descriptors. e) Relationship between the onset potentials of Cu‐free DACs@2D and the proposed descriptor for the CO_2_RR. Rectangles represent experimental values. MAE = mean absolute error. Reproduced with permission.^[^ ^27e^ ^]^ Copyright 2022, American Chemical Society.

For predicting adsorption energy, intrinsic atomic properties that can be obtained from the periodic table, handbooks or material databases are of great value; their use can improve the efficiency of catalyst discovery and make ML models more interpretable.^[^ ^27^ ^]^ Supported catalysts are known to perform well in many catalytic reactions; however, the complicated nature of the interfaces between support atoms and substrates poses a great challenge to rationally design excellent catalysts by traditional research methods. To overcome this challenge, Ren et al. combined DFT calculations and ML techniques to develop a simple and universal descriptor based on inherent atomic properties (electronegativity, electron type, and number). They employed this method to design 2D materials supporting dual‐atom electrocatalysts (DACs@2D) that possess superior activity and selectivity for CO_2_RR.^[^ ^27e^ ^]^ For a catalytic metal atom M interacting with the set of coordination atoms X, the proposed descriptor *φ* was expressed as *φ* = (*χ*_M_ + ∑*χ*_x_) + *N*_d/p_, in which the two terms (*χ*_M_ + ∑*χ*_x_) and *N*_d/p_ represent the coordination environment defined by electronegativities *χ* and the number of d or p electrons, respectively, of metal atom M (**Figure** **5**a). This descriptor actually quantifies the complicated interfacial effects operating in the DACs@2D system that ultimately determine the catalytic performance of the metal centers. Due to the conservation of orbital symmetry, d electrons dominate the binding strength between catalysts and intermediates related to CH_4_ or CH_3_OH production, while p electrons are the determining factor for the activity of CO_2_RR to HCOOH. Therefore, the descriptor *φ*_1_ (*φ*_1_ = (*χ*_M_ + ∑*χ*_x_) + *N*_d_) can well evaluate the activity of CO_2_RR to CH_4_ or CH_3_OH, corresponding to a volcano‐type curve by plotting the onset potential $U_{\mathrm{CH}4/\mathrm{CH}3\mathrm{OH}}^{\mathrm{onset}}$ as a function of *φ*_1_ (Figure 5b); the activity for HCOOH production can be evaluated well by using *φ*_2_ (*φ*_2_ = (*χ*_M_ + ∑*χ*_x_) + *N*_p_) as the descriptor, and the onset potential $U_{\mathrm{HCOOH}}^{\mathrm{onset}}$ proved to have a volcano‐type linear relationship with *φ*_2_ (Figure 5c). According to these linear relationships, a design or screening procedure for DACs@2D that produced excellent activity and product selectivity was established (Figure 5b). The procedure commenced by first calculating the values of *φ*_1_ and *φ*_2_ and their difference, then determining differential product selectivity according to whether their difference was larger or smaller than 6, and finally evaluating the activity for specific products according to the values of *φ*_1_ or *φ*_2_. Following this procedure, a fast design/screening to identify highly active DACs@2D with CO_2_RR product selectivity for predominately either CH_4_/CH_3_OH or HCOOH was achieved. Interestingly, although the descriptor *φ* was proposed based on Cu‐containing DACs@2D, it was also applicable to Cu‐free DACs@2D for CO_2_RR, as reflected by the accordance of data points of Cu‐free DACs@2D with the volcano relationship established by Cu‐containing DACs@2D (Figure 5c). More importantly, the descriptor *φ* is also applicable to other electrocatalytic reactions such as the N_2_ reduction (NRR) and O_2_ reduction (ORR) reactions. This study developed a simple and universal descriptor replacing experimentally unavailable adsorption energies to predict potentially useful supported catalysts for electrochemical reduction reactions.

### 3.2 Electronic and Structural Property Descriptors

**Figure 6:**
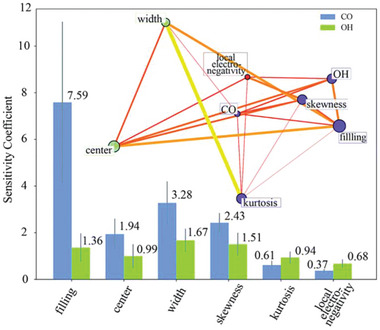
Feature importance scores for *CO and *OH adsorption energies based on sensitivity analysis of ANN models. The inset shows the linear dependence among input features and adsorption energies. Node size is proportional to the degree of linear dependence of one variable on the others. Reproduced with permission.^[^ ^29b^ ^]^ Copyright 2017, The Royal Society of Chemistry.

Descriptors based on intrinsic atomic properties usually fail to describe more complex systems, as the electronic and geometric structures peculiar to the active sites have to be considered. The d band chemisorption theory and its recent developments^[^ ^28^ ^]^ that relate electronic properties of active sites to adsorption energies of key intermediates have been one of the most successful descriptors for pristine transition metal surfaces and their alloys.^[^ ^29^ ^]^ Li et al. combined DFT‐calculated d‐band features of active sites and an ANN model to learn and predict adsorption energies of *CO and *OH on alloy surfaces for the rapid screening of bimetallic catalysts.^[^ ^29b^ ^]^ The d‐band features used include filling (zeroth moment up to the Fermi level), center (first moment relative to the Fermi level), width (square root of the second central moment), skewness (third standardized moment), and kurtosis (fourth standardized moment). In addition, local electronegativity was also selected to assess the contribution of sp‐electron density to adsorption energies. Once well‐trained, ANN models for *CO and *OH data sets led to root‐mean‐squared errors between predictions and DFT calculations for new bimetallic catalysts of about 0.2 eV, and suggest 3d‐metals, for example, Fe, Co and Ni, as promising additives in bimetallic catalysts for the methanol electro‐oxidation reaction. Feature importance analysis was carried out using normalized sensitivity coefficients, which reflect the degree of dependency of a target property on a given input feature. The normalized sensitivity coefficients for *CO and *OH adsorption energies with respect to six primary features are shown in **Figure** **6**. The results indicate that *CO adsorption has a strong dependence on d‐band features because CO has unoccupied 2π* molecular orbitals right above the Fermi level available for hybridization, while *OH adsorption has a strong dependence on sp‐band properties that are determined by local electronegativity. Existence of linear dependence between the six primary features and the *CO/*OH adsorption energies (Figure 6, inset) was affirmed by affinity propagation and graphical lasso algorithms,^[^ ^30^ ^]^ which confirmed that d‐states and sp‐states act as distinct governing factors of *CO and *OH adsorption on bimetallic catalyst surfaces.

**Figure 7:**
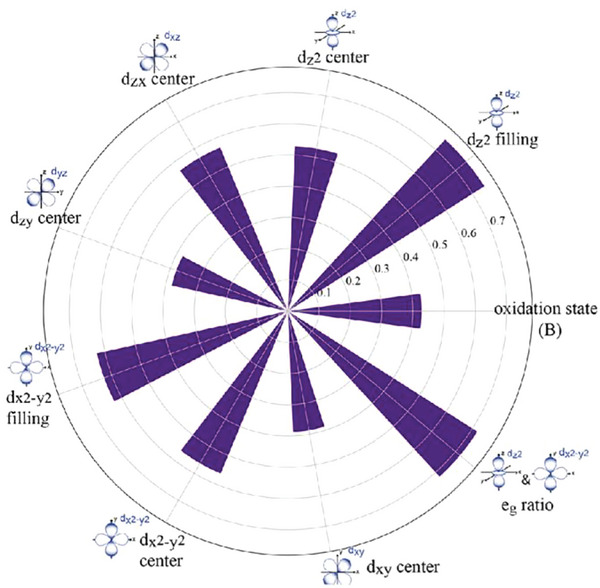
Polar distribution plots of the most informative descriptors with Kullback–Leibler divergence >0.4. Reproduced with permission.^[^ ^29d^ ^]^ Copyright 2020, American Chemical Society.

Although d band theory has been widely used for metal catalysts, it is less successful in predicting the catalytic activity of metal oxides, such as perovskite oxides, for which the occupancy of antibonding *e*_g_ orbitals has proven to be a good descriptor in the oxygen reduction reaction.^[^ ^31a^ ^]^ Li et al. developed an adaptive ML strategy to search high‐performance ABO_3_‐type perovskites for catalyzing OER and revealed that the *e*_g_ orbital characteristics of the metal B‐site are underlying factors that govern catalytic activity.^[^ ^29d^ ^]^ By learning the correlations between the selected descriptors (i.e., compositional and electronic structures) and the adsorption energies (i.e., of *O and *OH) of the ≈250 perovskites initially computed, a Gaussian process regression (GPR) model quickly estimated the adsorption energies of ≈4000 AA′B_2_O_6_ double perovskites, and finally singled out stable structures with promising OER activity. Then, Kullback–Leibler (KL) divergence analysis^[^ ^31b,c^ ^]^ was performed to understand physical factors governing the OER activity. A large KL divergence value indicates that the corresponding descriptor is informative and plays an important role in distinguishing the samples from two categorical classes. The results indicated that the electronic structure descriptors are more informative than the compositional descriptors in distinguishing the candidates with high and low OER activity. The topmost informative descriptors with KL divergence higher than 0.4 are shown in **Figure** **7**. It can be concluded that the *e*_g_ orbital (i.e., d_z2_, d_x2−y2_) properties, specifically the *e*_g_ electron occupancy including d_z2_ filling and d_x2−y2_ filling, are closely related to perovskite OER activity. From the molecular orbital point of view, this is because the d_z2_ orbital, one component of *e*_g_ orbitals, directly overlaps the p orbitals of oxygen intermediates at a superficial site. This study illustrates that electronic structure descriptors have advantages in disclosing the underlying physical factors that govern catalytic activity.

As different electronic characteristics are derived from different geometric structures, effective geometric descriptors can differentiate among the local environmental characteristics of various active sites, thus predicting adsorption energies and establishing structure–property relationships. Various effective geometric descriptors have been developed for use in heterogeneous catalysis research, such as bond length,^[^ ^32^ ^]^ rotational angle,^[^ ^33^ ^]^ Smooth Overlap of Atomic Positions (SOAP),^[^ ^34^ ^]^ Many‐Body Tensor Representation (MBTR),^[^ ^35^ ^]^ Atom‐Centered Symmetry Functions (ACSF),^[^ ^36^ ^]^ Coulomb Matrix (CM),^[^ ^37^ ^]^ coordination number^[^ ^38^ ^]^ and so on. Wexler et al. discovered that the Ni—Ni bond length is the most important descriptor among many other structural and electronic descriptors in determining hydrogen evolution reaction (HER) activity of Ni_3_P_2_(0001) under different doping concentrations.^[^ ^32a^ ^]^ Further experiments that applied mechanical pressure to compress and expand the Ni_3_‐hollow sites showed that the effects of mechanical pressure and chemical pressure via nonmetal doping are in excellent agreement, indicating that the local geometry of active sites can be more important than their electronic character for determining catalytic activity. MBTR and CM are global descriptors based on a tensor representation and coulomb repulsion, respectively. In contrast, SOAP and ACSF are local descriptors. The former represents the local environment around a central atom by determining rotationally‐invariant Gaussian‐smeared positions of neighboring atoms, while the latter expresses neighboring atomic distances and angular interactions by symmetry functions for each atom in a system. Jäger et al. analyzed the performance of the structural descriptors SOAP, MBTR, ACSF, and CM as applied to the prediction of hydrogen adsorption energy on the surface of nanoclusters including MoS_2_ and AuCu.^[^ ^39^ ^]^ SOAP performed significantly better than the others, so it can be a good choice for nanocluster adsorption energy predictions. Moreover, the authors found that the combination of SOAP with MBTR did not improve the predictive accuracy over SOAP alone, indicating that the local environment plays a dominant role in hydrogen adsorption. However, when dopants or defects were added to nanoclusters, the addition of global descriptors improved the learning. This indicates that metal identity becomes more difficult to describe as catalyst complexity increases.

**Figure 8:**
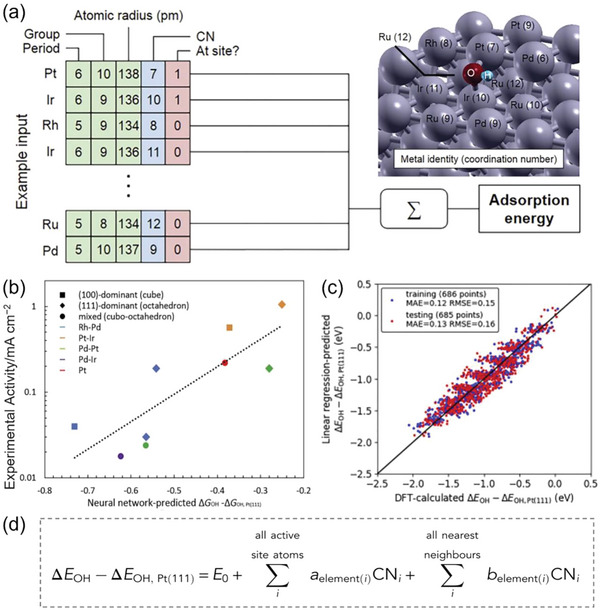
a) HEA catalyst example input features (green, blue and red indicate ligand, coordination and nearest‐neighbor descriptors, respectively), the NN layout, and the structure corresponding to the example input. b) Comparison between NN‐predicted adsorption energy and experimentally measured ORR activity. c) The NN model could be simplified to a linear scaling model at a slight loss of accuracy. d) The linear relationship between the adsorption energy and the CN descriptor, where CN*_i_* is the CN of the *i*th atom, and *E*_0_, *a*_element(_ *_i_*_)_ and *b*_element(_ *_i_*_)_ are parameters to be fitted. Reproduced with permission.^[^ ^38c^ ^]^ Copyright 2020, Cell Press.

High‐entropy alloys (HEA) are also very complex catalysts due to the immense chemical space that they span. Therefore, predicting catalytic performance of HEA catalysts is quite challenging due to the enormous number of active sites that may be described by the “ligand effect” and the “coordination effect.” As the former reflects surface electronic structure, its magnitude is dominated by metal element identity, while the magnitude of the latter—describing as it does surface geometric structures—is determined by the characteristics of crystal facets and defects. To account for both ligand and coordination effects on the catalytic performance of HEA catalysts (**Figure** **8**a), Lu et al. proposed a novel and simple representation of metal identity using elemental properties (i.e., period number, group number, atomic radius), coordination number (CN), and the proximity to adsorbate (i.e., at the active site or nearest neighbor).^[^ ^38c^ ^]^ When applied to the ORR, the above descriptors combined with a NN model can predict with high accuracy [the testing mean absolute error (MAE) and RMSE are 0.09 and 0.12 eV, respectively] the adsorption energies of OH*, a key ORR reaction intermediate, on IrPdPtRhRu HEA catalysts. Despite the challenges in bridging the theory‐experiment gap, general agreement was found between experimentally measured activity and NN‐predicted adsorption energy (Figure 8b). The authors also found that using only the simple CN descriptor can predict catalytic performance, implying that CN is the dominant influence on the adsorption energy. By simplifying the complex NN model to a linear scaling model at a slight loss of accuracy, a numerical relationship between CN and adsorption energy was obtained (Figure 8d). In this simplified model, element (*i*) refers to the element identity of *i*th atom in the IrPdPtRhRu catalyst. CN*_i_* is the coordination number of the *i*th atom, and *E*_0_ (intercept), *a*_element(_ *_i_*_)_, and *b*_element(_ *_i_*_)_ are parameters to be fitted. It shows that the contribution of each atom to the adsorption energy is weighted by both its CN and a factor specific to its elemental identity and its proximity to adsorbate. After performing least‐squares fitting using the same 50%/50% training/testing dataset split as in NN model, the testing MAE and RMSE are 0.13 and 0.16 eV (Figure 8c), respectively, slightly higher than those from NN. This confirmed the major contribution of CN between the active site and nearest neighbor atoms to the adsorption energy.

### 3.3 Multilevel Attention Mechanisms‐Identified Descriptors

The above works illustrate that both intrinsic atomic properties and electronic and geometric structures have been widely utilized as catalytic descriptors in theoretical ML models. The choice of appropriate descriptors usually requires expert experience with the studied systems, and the combination of these descriptors has proven to be a successful strategy to improve predictive accuracy.^[^ ^40^ ^]^ Beyond constructing effective descriptors through human intuition, the attention mechanism of deep learning models can help identify important features that have the potential to serve as effective descriptors for the prediction of target variables. Ma et al. have developed a multilevel attention graph convolution neural network (MA‐GCNN) that has been applied to predict the energies of hydroxyapatite nanoparticles^[^ ^41^ ^]^ and the quantum chemical properties of organic molecules.^[^ ^42^ ^]^ For a given molecule, the weights assigned to different effects of adjacent atoms on the central atom are called “attention.” In contrast to other attention algorithms that use “attention” at a single specific step, MA‐GCNN applies multilevel attention at every message‐passing step, gradually capturing the influence of different atomic nodes at each time step.

**Figure 9:**
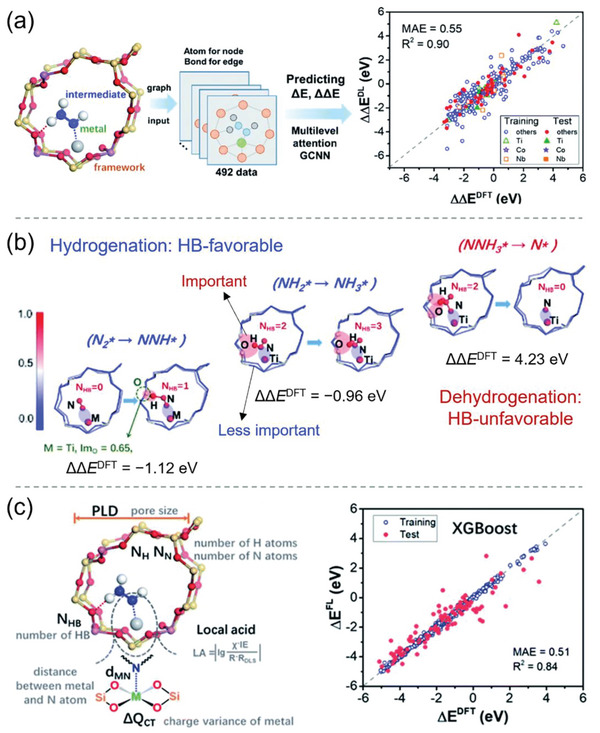
a) A flowchart of Δ*E* (relative energy to free N_2_) and ΔΔ*E* (energy difference between two successive steps) prediction by a multilevel attention graph convolutional neural network (GCNN) applied to NRR metal‐zeolite catalysts. b) The multilevel attention mechanism reveals that hydrogen bonding (HB) interactions favor the hydrogenation step but disfavor the dehydrogenation step. DFT calculated ΔΔ*E* values for these steps are given. Red and blue colors denote relative weights of 1 and 0, respectively. c) Descriptors used for predicting Δ*E* and the results predicted by the XGBoost model. MAE = mean absolute error; PLD = pore largest diameter; *N*_HB_ = number of hydrogen bonds. Reproduced with permission.^[^ ^43^ ^]^ Copyright 2022, The Royal Society of Chemistry.

By using MA‐GCNN, Gu et al. identified the important roles played by hydrogen bonding (HB) interactions and metal coordination (metal acidity) in predicting reaction energies of the NRR in metal‐zeolites, which motivated them to select HB features and local acidity (LA) as descriptors for constructing explainable ML models.^[^ ^43^ ^]^ As shown in **Figure** **9**a, the atom and bond of a metal‐zeolite can be regarded as the node and edge of a graph, respectively, and then a MA‐GCNN model was used to predict the energy changes (Δ*E*: the relative energy to free N_2_; ΔΔ*E*: energy difference between two successive steps) of the NRR process. The GCNN model provided good predictions with either training or test sets as inputs, with a MAE of 0.55 eV and a coefficient of determination (*R* ^2^) of 0.90. The results of the attention mechanism for the three main hydrogenation and dehydrogenation steps are shown in Figure 9b. Attention gave large weights (red color) to the metal centers (M, e.g., Ti), intermediates (NN*, NNH*, NH_2_*, NH_3_*, NNH_3_*, N*), and HB interactions between the H atoms of intermediates and O atoms of channels. Quantitative analysis showed that channel O atoms have relatively high importance values (Im_O_), for example, Im_O_ = 0.65 in Ti‐zeolite. By correlating the number of HBs (*N*_HB_) and the relative energy changes ΔΔ*E*, the effects of HB interaction were revealed. Specifically, the hydrogenation steps N_2_* → NNH* (ΔΔ*E* ^DFT^ = −1.12 eV) and NH_2_* → NH_3_* (ΔΔ*E* ^DFT^ = −0.96 eV) in Ti‐zeolite became more exothermic as the number of HBs increased from 0 to 1 and 2 to 3, suggesting that the hydrogenation steps are HB‐favorable. In contrast, the dehydrogenation step NNH_3_* → N* is energetically unfavorable, with ΔΔ*E* ^DFT^ = +4.23 eV. These results emphasize the important role of HB interactions in the NRR process. To capture the significant effect of metal atoms, the LA descriptor was defined as the combination of the electronegativity, first ionization energy, and atomic radius of the embedded metal (Figure 9c). By using the number of HBs, LA, geometric and other parameters (*d*_MN_: distance between the metal and N atom; PLD: pore largest diameter; *N*_H_ and *N*_N_: the number of H and N atoms in the intermediate) and a charge descriptor (Δ*Q*_CT_: the charge variance of the metal center), the reaction energies (Δ*E*) along the NRR pathway could be accurately predicted by the XGBoost model, with MAE of 0.51 eV and *R* ^2^ of 0.84. The predicted maximum energy inputs ΔΔ*E*_max_ were in qualitative agreement with the experimental NH_3_ yield, indicating the feasibility of the MA‐GCNN model for guiding the design of experiments. By employing SISSO (discussed in detail below), a linear equation between SISSO descriptors and relative energies was obtained, which confirmed the synergistic effect on the reaction energies between metal coordination and HB interactions. The LA descriptor has good transferability to metal‐containing and B‐doped 2D materials and other porous materials, such as metal–organic frameworks (MOFs) and covalent–organic frameworks (COFs).

### 3.4 SISSO Method‐Constructed Descriptors

Data‐driven descriptors constructed by the compressed sensing method SISSO (sure‐independence screening and sparsifying operator)^[^ ^44^ ^]^ have also shown effectiveness in predicting catalytic activity. Based on a combinatorial pool of features and mathematical operators, SISSO can handle high‐dimensional and nonlinear relationships and identify the best descriptor out of an immensity of even billions of candidates,^[^ ^45^ ^]^ an impossible task for either human intuition or linear models. In many cases, SISSO‐generated high‐dimensional descriptors can have much higher prediction accuracy than their low‐dimensional counterparts, while maintaining similar levels of interpretability.

**Figure 10:**
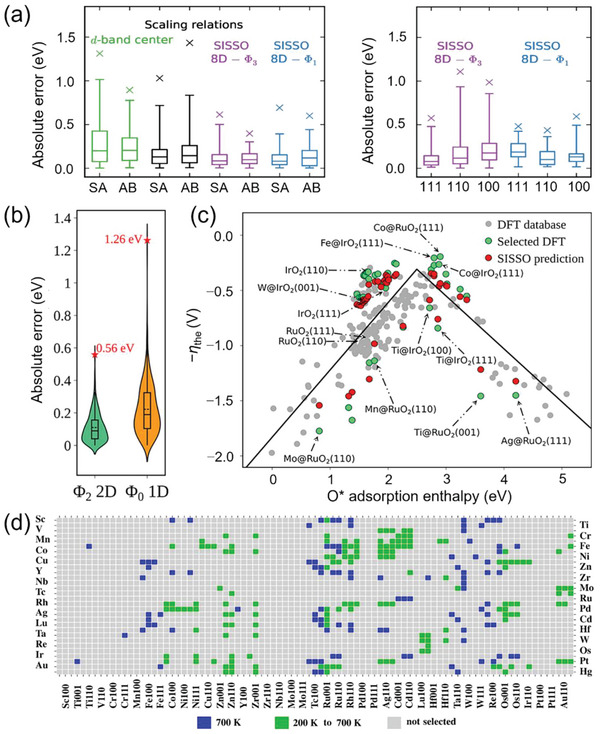
a) Box plots of the absolute errors for the d‐band center, scaling relations, and best SISSO descriptors. The upper and lower limits of the rectangles mark the 75th and 25th percentiles; the internal horizontal line marks the median, and the error bars mark the 99th and 1st percentiles. The crosses mark the maximum absolute errors. SA = single atom alloy; AB = bimetallic alloy. Reproduced with permission.^[^ ^45a^ ^]^ Copyright 2019, American Chemical Society. b) Violin plot of the distribution of absolute validation errors for the *Φ*2‐2D and *Φ*0‐1D descriptors. c) Negative theoretical overpotential *η*_the_ as a function of O* adsorption enthalpy. The black curve is the volcano predicted from the standard scaling relations. Selected DFT‐computed (green) and corresponding SISSO‐predicted (red) theoretical overpotentials are shown. Reproduced with permission.^[^ ^45b^ ^]^ Copyright 2021, American Chemical Society. d) High‐throughput screening of single‐atom‐alloy catalysts by SISSO. Promising candidates at different temperatures are highlighted. Vertical and horizontal axes display the guest atom type and the host metal surfaces, respectively. Reproduced with permission.^[^ ^47^ ^]^ Copyright 2021, Springer Nature.

By using the SISSO method with primary features related to a metal—its identity, bulk, surface, and adsorption site—Andersen et al. constructed new descriptors, expressed as nonlinear functions of a number of primary features, that predicted adsorption energies with better accuracy and generality than previous approaches such as scaling relations.^[^ ^45a^ ^]^ Comparison of the predictive ability of the d‐band center and scaling relations with the best SISSO descriptors on single‐atom (SA) and AB bimetallic alloys demonstrated great improvement of the new SISSO descriptors compared to the other two approaches (**Figure** **10**a, left). Note that the 8D‐*Φ*3 descriptor was identified using the alloy validation dataset alone, while the 8D‐*Φ*1 descriptor was identified by including 50% of the (111), (110) and (100) facet dataset to achieve a good compromise in accuracy between alloy composition and facets. Indeed, the much less complex 8D‐*Φ*1 descriptor has a better predictive performance when applied to low‐index fcc facets than the 8D‐*Φ*3 descriptor (Figure 10a, right)—indicating that the SISSO descriptor has good transferability across a wide range of structural motifs. More recent work has demonstrated that the SISSO method can also design good descriptors for adsorption enthalpies of OER intermediates on various facets of doped IrO_2_ and RuO_2_, for which the standard scaling relations (OOH* versus O*, OH* versus O*) in the form of simple correction terms cannot give reliable predictions.^[^ ^45b^ ^]^ The best SISSO descriptor 5D‐*Φ*3 based on only electronic and primary geometric features calculated assuming a clean surface performed better than the scaling approach in terms of accuracy and computational cost. By adding the O* adsorption enthalpy to the set of primary features, a less complex but more accurate 2D‐*Φ*2 descriptor was obtained, with a maximum absolute error (MAE) reaching 0.56 eV, while the 5D‐*Φ*3 and standard scaling relations yielded larger MAEs of 0.65 and 1.26 eV, respectively (Figure 10b). More importantly, the 2D‐*Φ*2 SISSO descriptor containing both O* adsorption enthalpy and features related to local charge transfer provided refined scaling relations; these correctly identified Co and Fe dopants as promising OER electrocatalysts in agreement with previous experimental work.^[^ ^46^ ^]^ The SISSO‐refined scaling relation reliably captured those data points that deviated significantly from the standard scaling relations‐derived volcano curve (Figure 10c). Compared to the actual DFT‐computed theoretical overpotential, the uncertainty of the SISSO‐derived overpotential is on average similar to the intrinsic DFT error of 0.2 V, as reflected by the closeness of the green (DFT) and red (SISSO) data points. Again combining DFT calculations and SISSO, Han et al. reported a fast yet reliable high‐throughput method for screening more than five thousand single‐atom‐alloy catalysts (SAACs) of hydrogenation reactions; this method reduced the computational time by at least a factor of one thousand compared with a pure DFT approach.^[^ ^47^ ^]^ Their screening criteria included the hydrogen binding energy, the H_2_ dissociation energy barrier, and the guest‐atom segregation energy evaluated in the presence of adsorbed hydrogen. Accurate predictions were obtained by SISSO‐derived descriptors that were constructed based on only 19 primary features of the host surfaces and guest single atoms. Besides correctly evaluating the performance of the experimentally‐tested SAACs, more than 200 yet unreported promising candidates were identified as having improved stability and activity compared to the catalysts in the original test set. As shown in Figure 10d, 160 SAACs (in green) were predicted to be both active and stable at 200 K, and 102 SAACs (in blue and green) were classified as promising candidates for hydrogenation reactions at a higher temperature (700 K).

SISSO‐constructed descriptors are usually complex analytic formulas, reflecting the complexity of the relationships between the primary features and the target properties. While potentially interpretable, SISSO by itself does not provide a straightforward way of evaluating the relative importance of different features for identifying desirable changes in target properties. However, the data‐mining method Subgroup Discovery (SGD) has proved useful for facilitating a physical understanding of SISSO descriptors. Given a data set and a target property, the SGD algorithm identifies local patterns that maximize or minimize a quality function and describes them as an intersection of simple inequalities involving a defined set of features and a set of adjacent data cluster borders (a1, a2,…), for example, “(feature1 < a1) AND (feature2 > a2) AND ….”^[^ ^48^ ^]^ From this, SGD identifies both the most important subgroups and the relevant primary features for a given target property. Through a qualitative analysis of complex SISSO descriptors by SGD, Han et al. revealed the actuating mechanisms for desirable changes in the target properties, for example, increasing the catalyst's stability and reducing the reaction barrier, in terms of basic features of the material.^[^ ^47^ ^]^

### 3.5 Spectral Descriptors

The above excellent works demonstrate the importance of predicting interactions between catalysts and adsorbates for catalyst design or screening. Considering the large range of catalyst types and adsorbates, exploration for efficient and general descriptors is a long‐pursued goal. An ideal descriptor should be a function involving electronic characteristics of both catalysts and adsorbates and contain information on spatial electronic distribution (and therefore must be a vector rather than a scalar), and last but not least, produce quantitative values accessible by both experimental measurement and theoretical computation. The descriptors usually employed, such as basic atomic properties, and electronic and geometric structures, hardly meet any of the aforementioned requirements.^[^ ^49^ ^]^

**Figure 11:**
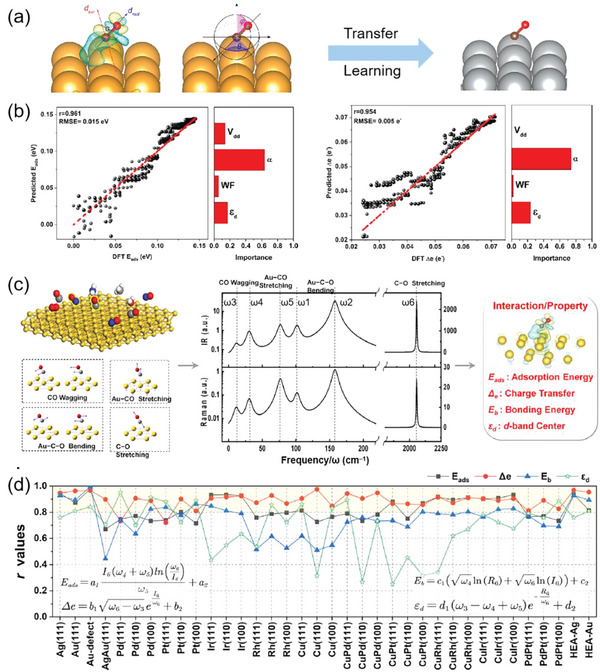
a) Illustration of the surface‐dipole (*d*_sur_) and molecule‐dipole (*d*_mol_) included angle *α* and the adsorption angles *φ* and *θ*. Transfer learning was achieved from CO@Au(111) to CO/Ag(111). b) Comparison of NN predictions and DFT calculations of *E*_ads_ and Δ*e* for CO@Ag(111), along with the corresponding feature importance analyses. Reproduced with permission.^[^ ^50^ ^]^ Copyright 2020, American Chemical Society. c) Using spectroscopy‐based features to predict surface–adsorbate interactions. Six vibrational modes (left), computed IR/Raman absorption spectra (middle) for CO adsorbed on Au(111), and target properties to be predicted (right). d) Pearson correlation coefficients of transfer learning applied to 34 new systems performed by the SISSO formulas as shown, trained from CO@Ag(111)/Au(111). See text for symbol definitions. Reproduced with permission.^[^ ^55^ ^]^ Copyright 2022, American Chemical Society.

Recently, our group proved the effectiveness of the electric dipole descriptor for predicting surface–adsorbate interactions including adsorption energy (*E*_ads_) and charge transfer (Δ*e*).^[^ ^50^ ^]^ The electric dipole moment parameters provide both quantitative and spatial information about electron distributions and are both experimentally measurable and theoretically computable. Using a NN ML technique, the *E*_ads_ and Δ*e* of CO and NO adsorption on an Au(111) surface were first investigated based on calculations of 10 000 adsorption configurations, generated by changing the adsorption angles *φ* (from 0 to 90°) and *θ* (from 0 to 180°) in small steps and then performing static calculations directly. Two descriptors were extracted from the surface‐dipole (*d*_sur_) and molecule‐dipole (*d*_mol_) moments, that is, the dipole–dipole interaction potential energy (*V*_dd_) and the angle *α* between *d*_sur_ and *d*_mol_ (**Figure** **11**a), quantities which have been shown to have a significant impact on charge/energy transfer.^[^ ^51^ ^]^ In addition, two descriptors widely used in catalysis research, work function (WF)^[^ ^52^ ^]^ and d‐band center (*ε*_d_),^[^ ^29c^ ^]^ were also included. The four selected descriptors more accurately predicted *E*_ads_ and Δ*e* of CO and NO adsorption on an Au(111) surface compared to DFT calculations. More importantly, the trained NN model using NO/CO@Au(111) can be applied to the interactions between molecules and additional substrates such as Au(001) and even Ag(111). Figure 11b shows the comparison of NN‐predicted and DFT‐calculated *E*_ads_ and Δ*e* values for CO adsorption on Ag(111). This comparison indicates that very accurate predictions with high Pearson correlation coefficients (*r*, 0.961 for *E*_ads_, and 0.954 for Δ*e*) and low root‐mean‐square errors (RMSE, 0.015 eV for *E*_ads_, and 0.005 e^−^ for Δ*e*) were achieved. Feature importance analysis revealed the effectiveness of the dipole‐related descriptor *α* for predicting *E*_ads_ and Δ*e* as it had the highest importance among the four descriptors.

In another, prior work, we found that the electronic spin moment is also a promising catalytic descriptor, as reflected by the linear relationship between catalytic activity and spin moment variation.^[^ ^53^ ^]^ Molecular dipoles are very difficult to measure in practice, but they have a strong association with vibrational spectra, such as infrared (IR) and Raman spectra, which can be measured experimentally, simulated theoretically and even predicted by ML tools.^[^ ^54^ ^]^ This inspires the utilization of vibrational spectral signals as catalytic descriptors. Indeed, we have demonstrated that surface–adsorbate interaction properties including adsorption energy and charge transfer can be quantitatively determined directly from IR and Raman spectroscopic signals of adsorbates.^[^ ^55^ ^]^ As shown in Figure 11c, six vibrational modes most relevant to CO adsorption (two bending modes *ω*_1_ and *ω*_2_, two wagging modes *ω*_3_ and *ω*_4_, one weak metal–C stretching mode *ω*_5_, and one strong C—O stretching mode *ω*_6_) and their corresponding IR/Raman intensities (*I* and *R*) were selected as the input features to describe target properties including *E*_ads_, Δ*e*, C—O bond energy (*E*_b_), and the d‐band center of the metal surface (*ε*_d_). Application of these 18 vibrational spectral features combined with ML extra‐trees regression (ETR) was used to accurately predict *E*_ads_, Δ*e*, *E*_b,_ and *ε*_d_ for the CO@Au(111)/Ag(111) system. Moreover, by employing SISSO, the machine‐learned spectrum–property relationships can be described by mathematical formulas, with adsorbate spectral features being the variables. Interestingly, these vibrational feature‐based formulas have generalizability to a series of new surface–adsorbate systems, including new metals, binary alloys and HEAs, with excellent predictive ability, typically with *r* values greater than 0.8 and many exceeding 0.9 (Figure 11d). It was also found that these spectroscopy‐based formulas allow the separation of contributions from substrate and adsorbate: the variables *I*, *R* and *ω* are adsorbate spectral signals, while the parameters *a*, *b*, *c* and *d* are constants related to intrinsic characteristics of the substrate. The development of spectral features as catalytic descriptors to establish quantitative spectrum–property relationships opens a new avenue for investigations of catalytic activity, circumventing the difficulties in learning detailed geometric structures of complex catalysts.

## 4 Descriptors for Combining Theoretical and Experimental Data in Machine Learning

Currently, ML has more applications in theoretical catalysis compared to experimental science because a large uniform dataset can be more readily obtained from theoretical calculations. Although high‐throughput experimentation can provide large datasets, these experiments are time‐consuming and expensive. Therefore, ingenious utilization of the smallest dataset capable of optimizing and discovering new catalysts is being actively studied. Karim et al. generated a uniform 36‐sample experimental dataset using high‐throughput synthesis and activity measurements and then employed gradient boosting regression (GBR) and support vector regression (SVR) models to correlate catalyst synthesis conditions with ORR activity. The ML models provided better synthesis parameters for their next batch of experiments, which identified a new catalyst with much higher ORR activity than that captured in the original dataset.^[^ ^56^ ^]^ Williams et al. also reported a framework incorporating ML regression algorithms with high‐throughput experiments to discover inexpensive catalysts for ammonia decomposition.^[^ ^57^ ^]^ Interestingly, by using a small initial experimental data set composed of only three catalyst compositions, they discovered a new catalyst—3,1,12RuYK/Al_2_O_3_ (3 wt% Ru, 1 wt% Y, and 12 wt% K)—that greatly outperformed the top‐performing Ru‐based catalysts reported in the literature. These results show that a ML model combined with appropriate descriptors can extract trends and rules from relatively small data sets, especially when the data sets are generated by autonomous experimentation in a uniform and bias‐free way.

**Figure 12:**
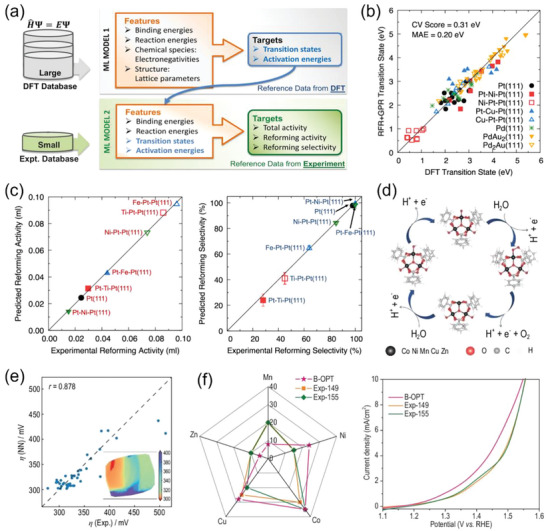
a) Flowchart of an approach consisting of two ML models, Models 1 and 2. b) Comparison of predicted transition state energies calculated by ML Model 1 versus DFT. c) ML model 2 predicted and experimental ethanol reforming activity and selectivity. The plotted data points in (b) and (c) were obtained from leave‐one‐out cross‐validation (CV). Reproduced with permission.^[^ ^58^ ^]^ Copyright 2020, American Chemical Society. d) Simulated OER reaction path. e) NN‐predicted compared to experimentally measured overpotentials. Inset: dimensionality reduction plot generated by principal component analysis (PCA) for predicted overpotentials of all 20 000 samples. f) Kiviat diagram of composition ratios and polarization curves of the optimal sample (B‐OPT) suggested by the Bayesian model and the best experimental samples (Exp‐149/155). Reproduced with permission.^[^ ^59^ ^]^ Copyright 2022, China Science Publishing &Media Ltd.

In addition to relying only on small experimental datasets, incorporating additional extensive computational data derived from suitable descriptors is preferable, as this strategy can cover a much broader catalytic space and enable the understanding of atomic‐level factors that control experimentally measured catalytic performance. Artrith et al. demonstrated that the combination of ML and DFT calculations can be employed to interpret experimental activity and selectivity and to predict the catalytic performance of additional catalysts for reforming ethanol to produce CO and H_2_.^[^ ^58^ ^]^ They first constructed ML model 1 for the prediction of ethanol decomposition transition‐state energies from thermochemical reaction energies using complex random forest regression (RFR) and Gaussian process regression (GPR). Then, these predicted transition‐state energies were entered into their ML model 2 that was trained on catalytic activities and selectivities comprised of only 7 experimental datapoints using simple linear regression (**Figure** **12**a). Results predicted by leave‐one‐out cross‐validation (CV) by each of the two models (Figure 12b,c) indicate that the introduction of accurately‐predicted transition‐state energies leads to the very good predictive accuracy of model 2. Moreover, linear model 2 revealed that both activity and selectivity are strongly positively correlated with C—C bond‐scission transition‐state energy, suggesting that this reaction is a key step and favors the competing ethanol decomposition reactions such as methane production. Beyond understanding the reaction mechanisms, both models together allow the prediction of catalytic activity and selectivity directly from principal chemical properties and features that can be efficiently determined with high‐throughput DFT calculations. The success of this work is probably because the knowledge of reaction energies and kinetic activation energies of different reaction pathways is sufficient to predict both activity and selectivity. However, the acquisition of activation energy is computationally demanding, making it not well suited for high‐throughput calculations.

Recently, our group has demonstrated that by using adsorption energies and charge transfer as intermediate descriptors, combined ML models allow the prediction of overpotentials for the OER catalyzed by high‐entropy materials. Results of these models suggested an optimal composition ratio that performed far better than any resulting from human intuition.^[^ ^59^ ^]^ The computational dataset consisted of about 20 000 structures generated by molecular dynamics simulations;^[^ ^60^ ^]^ the corresponding DFT‐calculated catalytic properties included Gibbs free energy changes of key intermediates and charge transfer. Using metal composition ratios as descriptors, these catalytic properties were accurately predicted by a NN model trained on the extensive DFT data. Note that the direct calculation of overpotentials based on the reaction mechanism shown in Figure 12d resulted in systematic errors of the absolute values compared with experimental overpotentials. However, when adding the accurately‐predicted catalytic properties as descriptors, the measured overpotentials of 207 robotically‐executed experiments were matched reasonably closely, with a Pearson correlation coefficient of 0.878 (Figure 12e). Therefore, overpotentials of all 20 000 structures were rapidly predicted based on metal composition ratios (the inset of Figure 12e); the optimal composition ratio predicted by Bayesian optimization (an iterative response surface‐based global optimization algorithm)^[^ ^61^ ^]^ over the huge search space (553 401 options) differed greatly from the best samples obtained by the trial‐and‐error experiments (Figure 12f, left). The Bayesian optimization‐suggested sample (B‐OPT) was experimentally validated to have better catalytic performance than the best‐performing samples out of the 207 experiments (Figure 12f, right). This study showed that a small experimental dataset can correct the absolute error of theoretical calculations, while a computational dataset can expand the exploratory scope of experiments. This is particularly advantageous when catalysts are difficult to synthesize or characterize under operating conditions. Glossary of machine‐language terms can be found in the Supporting Information.

## 5 Conclusions and Perspectives

Machine learning techniques have been increasingly applied to catalysis research and, due to their new powerful learning and predictive abilities, have solved the many bottleneck‐creating problems encountered in traditional trial‐and‐error experiments and theoretical simulations. The selection of appropriate input features (descriptors) is crucial to the improvement of predictive accuracy and the understanding of catalytic mechanisms. In this review, we discussed the tactics for the utilization and extraction of descriptors in experimental and theoretical ML models. For ML models based on experimental data, comprehensively describing a catalytic reaction using various dimensions and types of descriptors is a basic strategy. When available data are limited, performing iterative learning with a variety of descriptors can extract key information for the discovery of new catalysts. Additionally, collecting a large amount of data from high‐throughput experimentation or published papers provides another way to identify effective descriptors for the rational design of catalysts. For ML models based on theoretical simulations, rationally selected intrinsic atomic properties, and electronic and geometric structures such as d band features and coordination number can serve as effective descriptors for the prediction of adsorption energies of key reaction intermediates. Molecular dipole parameters, and especially vibrational spectral signals that can be experimentally measured and theoretically simulated, have been demonstrated to be promising catalytic descriptors, opening a new window for catalysis research. The multilevel attention mechanism of deep learning provides a feasible method for identifying novel descriptors for the prediction of reaction energies. Further, one can use SISSO to generate an immensity of candidate descriptors and select the most relevant ones for handling high‐dimensional and nonlinear relationships. Finally, we discussed a promising research paradigm that combines theoretical and experimental ML models through suitable intermediate descriptors. This paradigm makes use of the respective advantages of experimental and computational datasets, so that the small and expensive but accurate data of the former are combined with the rough but cheap data generated by the latter.

Despite these achievements, further efforts should be made in many areas to unleash the power of descriptors and ML tools in the field of catalysis. First, the development of universal and interpretable descriptors that are easy to calculate or measure, and contain key information about catalysts and adsorbates and their interactions is still in its infancy. In the future, integrated and comprehensive descriptors that combine basic properties of elements with electronic and geometric structures are worthy of investigation, as a single type of descriptor usually has limitations and cannot describe the various materials and reactions completely. Moreover, spectroscopic descriptors, including numeric features and spectral images that can be considered as a package of geometric and electronic characteristics, also deserve attention. Second, the prediction of realistic catalyst compositions based on theoretical simulations remains a great challenge, because calculations for a catalytic reaction at this stage are usually performed assuming vacuum conditions, thus poorly modeling the realistic catalytic environment. The research paradigm of combining computational and experimental datasets provides a solution for this issue, but this method is still underexplored in terms of intermediate descriptors and ML models. Going further, use of ML‐based large‐scale atomic simulations that rely on evaluation of the potential energy surface is also a promising approach for predicting catalytic system performance under realistic conditions.^[^ ^62^ ^]^ However, there are still difficulties in constructing ML potentials for complex multielement systems, and the predictive accuracy is restricted by the level of DFT employed. Third, expansion of existing material databases and establishment of new comprehensive catalysis databases should be considered for facilitating the acquisition of data points and descriptors. We believe that in the future, one could construct an ML model based on sufficient information of descriptors and target properties contained in shared databases before experimentation, and then calibrate the ML model using a few accurate and consistent experimental results, finally leading to accurate prediction of actual catalytic performance over the huge, entire catalytic space.

## Conflict of Interest

The authors declare no conflict of interest.

## Supporting information

Supporting Information
