# Supplementary material for: Machine Learning Descriptors for Data‐Driven Catalysis Study
Source: Adv Sci (Weinh). 2023 May 16;10(22):2301020. doi: 10.1002/advs.202301020 (PMC10401178; doi:10.1002/advs.202301020)
Supplement: Supplementary file 1 — Supporting Information [file ADVS-10-2301020-s001.pdf]

ADVANCED SCIENCE
Open Access

Supporting Information

for *Adv. Sci.*, DOI 10.1002/advs.202301020

Machine Learning Descriptors for Data-Driven Catalysis Study

Li-Hui Mou, TianTian Han, Pieter E. S. Smith, Edward Sharman and Jun Jiang**

Supporting Information

Machine Learning Descriptors for Data-driven Catalysis Study

Li-Hui Mou, TianTian Han, Pieter E. S. Smith, Edward Sharman, and Jun Jiang**

Glossary of machine-language terms

One-hot vector	A binary-valued vector with one element set to 1, and all the remaining elements set to 0.
Attention mechanism	Attention is a technique used in artificial neural networks that enhances some parts of the input data while diminishing other parts, thus devoting more focus to the typically small, but important, parts of the data.
Random Forest Regression	Random forest combines the output of multiple decision trees to reach a single result. Each tree in an ensemble is comprised of learning over a data sample drawn from a training set with replacement. Of that training sample, one-third is set aside as test data. Feature bagging adds more diversity to the dataset and reduces the correlation among decision trees. For a regression task, such as determining the linear dependence of one variable on another, the outputs of the individual decision trees are averaged to give the random forest regression result.
Extra Trees Regression	Extra Trees Regression is similar to Random Forest Regression, except that the entire training set is used without replacement, and the decision tree nodes are randomized instead of being optimized.
Gaussian Process Regression	In Gaussian Process Regression GPR), observed data points are used to fit a function representing these data points, and then the function is used to make predictions at new data points. For a given set of observed data points, there are an infinite number of possible functions that fit them. In GPR, Gaussian (i.e. normally-distributed) processes are employed to conduct regression by defining a joint distribution over this infinite number of functions that is used to discover an optimally-predictive function.
Feature Importance	Feature importance indicates how much each feature contributes to the model prediction and is used as a tool for ML model interpretability. It assigns scores to input features

	based on their importance to predict the output.
MFF = molecular fragment featurization	A modified method of molecular characterization utilizing substructure and similarity searching developed specifically for structure-activity modeling.
SISSO	The Sure-Independence Screening and Sparsifying Operator is a catalyst design generator based on the *compressed sensing* method used in signal processing to extract information from sparse signals. It works for catalyst design because out of the immense number of materials combinations that could form catalysts, only a very few meet the desired activity and stability criteria.
SYBA Score	Scores of Bayesian Accessibility is a statistical method for estimating how hard an organic molecule might be to synthesize. It is based on how frequently the molecular fragments that make up a molecule appear in a large database of molecules, *i.e.*, the more frequent the occurrence of its fragments, the more easily a molecule should be to synthsize.
Voting Regressor	A Voting Regressor aggregates the predictions of multiple base models and makes a final prediction. Voting Regressors can be useful when applied to a set of equally well performing models in order to balance out their individual weaknesses.
Cross-validation (CV)	Cross-validation is a statistical technique used to evaluate the performance of a machine learning model. It involves dividing the data into two sets, a training set and a testing set. The model is trained on the training set and then tested on the testing set.
Principal Component Analysis (PCA)	PCA works by transforming the original data into a new set of variables, called principal components, which are linear combinations of the original variables. These principal components are ordered in such a way that the first component explains the most variation in the data, and each subsequent component explains as much of the remaining variation as possible.
